# Platinum Resistance in Ovarian Cancer: Role of DNA Repair

**DOI:** 10.3390/cancers11010119

**Published:** 2019-01-20

**Authors:** Giovanna Damia, Massimo Broggini

**Affiliations:** Laboratory of Molecular Pharmacology, Istituto di Ricerche Farmacologiche Mario Negri IRCCS, via G. La Masa 19, 20156 Milan, Italy

**Keywords:** ovarian cancer, nucleotide excision repair, DNA damage response, DNA repair, homologous recombination, DNA damaging agents, cisplatin, DNA polymorphisms, gene mutations, drug resistance

## Abstract

Epithelial ovarian cancer (EOC) is the most lethal gynecological cancer. It is initially responsive to cisplatin and carboplatin, two DNA damaging agents used in first line therapy. However, almost invariably, patients relapse with a tumor resistant to subsequent treatment with platinum containing drugs. Several mechanisms associated with the development of acquired drug resistance have been reported. Here we focused our attention on DNA repair mechanisms, which are fundamental for recognition and removal of platinum adducts and hence for the ability of these drugs to exert their activity. We analyzed the major DNA repair pathways potentially involved in drug resistance, detailing gene mutation, duplication or deletion as well as polymorphisms as potential biomarkers for drug resistance development. We dissected potential ways to overcome DNA repair-associated drug resistance thanks to the development of new combinations and/or drugs directly targeting DNA repair proteins or taking advantage of the vulnerability arising from DNA repair defects in EOCs.

## 1. Introduction

The development of drug resistance represents one of the major obstacles to the cure of cancer. In fact, in many instances, tumors that are initially responsive to a given drug will relapse with a tumor no longer sensitive to the initial drug. Several mechanisms contribute to the development of drug resistance, including tumor heterogeneity, reduced drug concentration to the target, alteration in drug target structure, increased repair of the lesions induced. Depending on the drug and tumor under investigation, one or more mechanisms can take place. A better understanding of the different mechanisms of resistance along with the development of suitable models to find ways to overcome resistance is mandatory to improve the outcome for cancer patients.

Platinum containing drugs (cisplatin, carboplatin, and oxaliplatin) are very active agents widely used for the treatment of different malignancies (i.e., testicular germ line, ovarian, lung, head and neck, colon). Specifically, platinum-taxanes doublet represents the gold standard treatment (in both adjuvant and neo-adjuvant setting) in epithelial ovarian cancer (EOC). Epithelial ovarian cancer is one of the most chemo-responsive tumors responding well to both cisplatin and carboplatin, although almost invariably after an initial response, patients will relapse with platinum resistant tumors. Resistance to platinum-containing drugs has been associated to several mechanisms, including alteration in drug efflux (through for example the modulation of the copper transporter CTR-1 expression) [1,2,3,4,5], alteration in intracellular proteins able to bind and sequester Pt (as for example GSH) [6,7,8,9], and altered expression of pro-survival or anti-survival proteins [10,11,12,13]. Considering that the main target of platinum drugs is DNA, the sensitivity/resistance to these drugs is affected/modulated by the ability of cells to recognize and repair the DNA drug induced damages. Specifically, there is strong preclinical evidence suggesting how the presence or absence of a specific DNA repair pathway (due to mutations, deletion or epigenetic changes of genes involved in DNA repair) is associated with sensitivity/resistance to platinum drugs [14,15,16,17,18].

We will here focus on DNA repair pathways as possible mechanism of resistance to a platinum-based therapy in ovarian cancer. In addition, the importance of predictive biomarkers of response to platinum will be described with the aim to possibly identify patients that will not respond to therapy and could eventually be re-directed to alterative therapeutic strategies.

## 2. Clinical Presentation and Management of Ovarian Cancer

Epithelial ovarian cancer represents one of the major causes of cancer death among women and certainly the most lethal gynecological cancer [19]. Epithelial ovarian cancer tumor is generally diagnosed at late stage (Federation of Gynecologists and Obstetricians (FIGO) stage III/IV) when the tumor is disseminated throughout the peritoneal cavity, limiting the potential benefit of debulking surgery. In fact, ovarian tumors present with metastases that infiltrate the omentum, peritoneum, diaphragm, and the Glissonian capsule and are associated with abdominal effusion (ascites). Epithelial ovarian cancer is not a single disease but comprises different histological entities (e.g., high-grade serous, endometrioid, mucinous, clear cell and low grade serous) with different biological, molecular, and clinical characteristics. Yet to be defined is also the cell of origin of these different entities, as both fallopian tube and ovary has been advocated as site of tumor development [20,21]. In spite of the evidence suggesting that the different types of EOC represent unique entities, they are still treated with a similar approach consisting of a de-bulking surgery followed by adjuvant chemotherapy. The latter comprises a platinum containing (mainly cisplatin and carboplatin)-taxanes doublet, which has not changed over the last three decades, with the aim to eliminate all the remaining micro-metastases. Platinum agents were in fact introduced in the late 1970s when different clinical studies reported that cisplatin caused a double in overall response rates and in complete responses as compared to non-platinum containing schedules [22,23]. Nevertheless, even if 80% of EOC patients respond initially to this first-line therapy, almost 80% of those responding cases will recur with resistant disease and the choice of second line therapy remains empirical, mainly based on the time lag between the end of chemotherapy and first relapse, defined as platinum-free interval (PFI) [24].

In the last decade, the molecular characterization of EOC has revealed that more than 50% of high-grade serous ovarian cancers (HGSOCs), representing 80% of EOCs, have a defect in homologous recombination (HR) repair, underlying the extreme sensitivity of this tumor type to platinum drugs [25]. Many studies, including TGCA (The Cancer Genomic Atlas) [26], have demonstrated that HGSOC is characterized by a defect in HR by genetic and epigenetic alterations in the genes involved in the pathway, including *BRCA 1* and *2* (for a recent review see Reference [25]). Not only does this underlying defect support the extreme sensitivity to platinum agents, but it also allows the clinical implementation of targeted therapy with Poly (ADP-ribose) polymerase inhibitors (PARPi). In fact, this class of agents has been clearly shown in preclinical studies to be extremely active in cellular systems deficient in HR repair by a synthetic lethality basis and such activity has been validated in clinical trials in *BRCA1/2* mutation carriers with ovarian carcinomas [27]. The PARPi olaparib, rucaparib, and niraparib have been recently approved in the US and Europe for clinical use against platinum-sensitive ovarian cancers, as they were shown to increase the progression free survival; however, no data on overall survival (OS) are available yet [28,29,30]. Recent data suggest that these compounds are also active in wild type *BRCA1/2* tumors [31].

## 3. DNA Repair Systems

Combinations containing platinum-based drugs, mainly cisplatin and carboplatin, represent the first line treatment for EOC. The mechanism of action of both cisplatin and carboplatin involves their interaction with DNA and the formation of mono-adducts, mainly covalently interacting with N7 of guanine. This mono-adduct then evolves, through a second covalent binding, to a DNA crosslink, which can be on the same DNA strand (i.e., intrastrand crosslinks representing the most abundant platinum adducts) or on the opposite strand (i.e., interstrand crosslinks which are responsible for the drug antitumor activity). The major differences between cisplatin and carboplatin are in the kinetics of mono-adduct and mono-adduct to cross-links formation, due to the different aquation rates and steric hindrance [32,33]. If these lesions are not repaired (either in the form of DNA mono-adduct or in the form of DNA crosslink) the consequence is a block of DNA synthesis and transcription; in addition, the replication fork delay can progress and completely destabilize DNA synthesis with activation of a replicative stress response. In more recent years, the molecular definition of these processes and of the proteins involved have defined their role also in chemo-resistance, including to platinum agents [34].

As a general rule, the presence of DNA lesions activates a DNA damage response (DDR) with the activation of cellular pathways leading to a slow down or interruption of DNA synthesis, a block of the cell cycle, and activation of repair pathways [35]. These pathways are part of the DDR and have a key role in the maintenance of genome integrity [36,37]. Genomic instability has been reported to be a hallmark of cancer [38,39]. Indeed, defects in DDR are associated with an increased risk of developing cancer during life time. Briefly, the first step of DDR consists in the recognition of the damage by “sensors” proteins that identify DNA structures induced by DNA damaging agents and replication stress. The master sensors are ATM (ataxia-telangiectasia mutated), ATR (ATM- and Rad3-Related), and DNA-PKs (DNA-dependent protein kinase). These are large serine/threonine kinases members of the phosphatidylinositol-3-kinase-like kinase family (PIKKs) and orchestrate a large network of cellular processes to maintain genomic integrity with distinct specificities and functions [40,41]. The ATM kinase is primarily activated by double-stranded DNA breaks (DSBs) and it leads to the phosphorylation of a number of substrates, such as BRCA1, CHK2, and p53, mediating DNA repair, cell-cycle arrest, and apoptosis. The ATR kinase, essential for the survival of proliferating cells because it monitors replication fork progression, responds to a broad spectrum of DNA damages, including single-stranded DNA breaks (SSBs) and a variety of DNA lesions that interfere with replication [42]. It phosphorylates substrates such as BRCA1, CHK1, p53, and RAD17 that, in turn, mediate inhibition of DNA replication and promote DNA repair. Finally, DNA-PKs regulate a smaller number of targets and play a role primarily in non-homologous end joining (NHEJ) [43,44].

**Figure 1:**
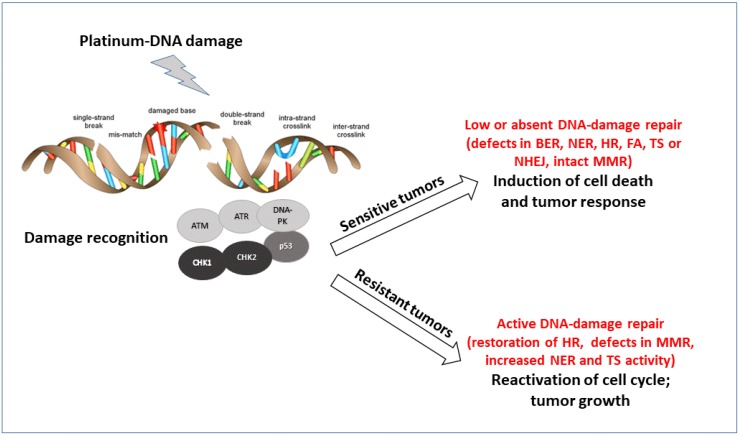
Schematic representation of the major pathways involved in DNA damage recognition and repair of platinum DNA damage. The major proteins acting as sensors and effectors of the DNA damage are reported. According to the tumor cell ability to repair the damage, cells will survive and acquired resistance to treatment or not repair and die.

There are six main different DNA repair pathways: mismatch repair (MMR), base excision repair (BER), nucleotide excision repair (NER), homologous recombination (HR), non-homologous end joining (NHEJ), and Fanconi Anemia (FA) (reviewed by Curtin [42]). The coordinated interplay among these different pathways results in repair and cell survival with no fixed DNA damage, low levels of repair, and accumulation of DNA damage that cells can tolerate but might predispose to the development of several pathologies, including tumors, or activation of cell death when DNA damage is too high to be repaired. Alteration (upregulation and downregulation) in these pathways contribute to the sensitivity and resistance to platinum agents (Figure 1). The detailed description of these pathways is beyond the scope of this manuscript [45,46], but it is worth saying that even if each DNA repair pathway is generally activated by specific DNA lesions, some redundancy among the different pathways exists. The lack of a specific repair can be associated with the upregulation of the other. Even if cells with a specific DNA repair deficiency will try to repair the DNA damage with the remaining DNA repair pathways, most of these cells will be, by definition, extremely sensitive to agents causing DNA damage repaired by that specific repair pathway or to those agents in synthetic lethality with that specific defect [47].

Almost all the major DNA repair mechanisms can participate in the removal of cisplatin adducts. In particular, several evidences support a pivotal role of NER, MMR, BER, HR, NHEJ, and FA pathways (Table 1). While the intra-strand crosslinks cause a distortion of the DNA double helix and activate mainly the NER pathway, the inter-strand cross-links rely on the coordinated interaction among translational synthesis, HR, NER, and FA pathways [48]. Briefly, NER is a highly conserved and versatile pathway used to remove “bulky lesions” (i.e., the ones caused by UV light), which distort the DNA double helix and has been involved in the repair of intra- and inter-strand crosslinks (ICLs), including the ones caused by platinum drugs. Two major NER pathways can be recognized: the transcription coupled repair (TCR) and the global genome repair (GGR); the former is a highly specific and efficient system that detects and removes the DNA damage that blocks the progression of RNA polymerase II in actively transcribed genes, while the latter is a slow process that inspects the entire genome. The initial steps involve the recognition of DNA damage by proteins (XPA, XPC, RPA) following by the unwinding of DNA by the helicase XPD and XPB. The DNA around the damaged site is then cleaved by the XPG 3′ and XPF–ERCC1 5′ nucleases. A DNA polymerase re-synthetizes the DNA sequence on the complementary strand [49,50]. Homologous recombination is an error free mechanism for the repair of DSBs or stalled replication forks occurring in S and G2 phases of the cell cycle, caused by IR, ROS, and some antineoplastic drugs, including platinum-based drugs. Homologous recombination uses the sister chromatid as a template to repair the DSB. When the break is recognized, BRCA1 mediates the recruitment of the MRN complex. The ATM kinase activates MRN components by phosphorylation and the complex resects the 3′ strand ends on either side of the DSB, the single strand DNA is unwound, and the 3′ strand from the damaged chromosome invades into the sister chromosome with the involvement of BRCA2 and RAD51. The damaged 3′ end is then extended using complimentary sequence by a DNA polymerase [51,52]. The FA pathway is particularly important for the removal of intra-strand cross-link. This pathway promotes the monoubititination of the FA complementation group D2 (FANCD2)–FA complementation group I (FANCI) heterodimer, activating the DNA damage response. The FA pathways not only ensures an efficient DNA damage repair through homologous recombination, but also coordinates DNA replication and fine-tunes mitotic checkpoints for an error-free chromosome segregation. Loss of the FA pathway by mutation in FA genes renders cells hypersensitive to DNA inter-strand crosslinking agents, including platinum drugs [53].

To better understand the role of these DNA repair pathways in the mechanism of action of platinum drugs, we will briefly summarize their importance in cellular response to these agents. Detailed in vitro studies, using isogenic systems, indicated that NER defects are associated with an extreme sensitivity (more than a 100-fold increase as regards parental wild-type cells) to cisplatin. In particular, two components of the NER, excision repair cross-complementing 1 (ERCC1) and Xeroderma Pigmentosum, Complementation Group F (XPF), are those mostly influencing platinum-DNA adducts removal [14]. In contrast to NER, defects of MMR are associated with resistance to both cisplatin and carboplatin [54,55,56], likely due to the ability of MMR proteins to detect the lesions and to activate apoptotic processes [57,58]. For BER, little evidence linking this repair system to platinum containing drugs sensitivity has been reported [59,60,61]. Homologous recombination and FA have also been shown to be important for the cellular response to platinum agents as cells deficient in these pathways are extremely sensitivity to them [62,63]. Finally, other proteins and pathways participating in the DNA damage response have been involved in cisplatin/carboplatin activity, such as ATR and ATM proteins (DNA damage sensors) and checkpoint proteins CHK1 and CHK2 [64,65]. Recently, the role of CDK12 in master regulation of DNA repair gene transcription, as outlined in a recent review [66], and in modulating sensitivity to PARP inhibitors and platinum agents has been displayed.

## 4. Alterations in DNA Repair in Ovarian Cancer and Their Prognostic/Predictive Value

As already stated, genetic data suggest that 50% of HGSOC exhibits defective DNA repair by inactivation of HR due to germline and somatic mutations in *BRCA1* (11%), *BRCA2* (9%), promoter hypermethylation of *BRCA1* (10%) and *RAD51C* (2%), and mutations in *FA* and core *RAD* genes (3.5%) [25]. Other molecular alterations have been correlated with a HGSOC HR deficiency. Specifically, *CDK12* mutations or amplification are present in approximately 4% of cases. It has been reported that CDK12 is a transcription-dependent kinase with a specific role in the transcription of long genes, including DNA repair genes. Preclinical evidence suggests that knock-down of this kinase sensitizes cells to both PARPi and platinum agents due to downregulation of genes involved in HR. Mutations interfering with its catalytic activity have been reported in ovarian cancers and likely to account for the HR deficiency [66,67]. Homologous recombination deficiency could also result from *PTEN* homozygous loss, reported in about 7% of HGSOC and *EMSY* amplification in 6% of tumors. In the former case, the proposed mechanism of HR deficiency induction is the transcriptional downregulation of RAD51, while the exact role of EMSY is still to be defined [25].

The *BRCA1* and *BRCA2* germline/somatic mutations are considered predictive biomarkers of response to PARP inhibitors, although these tumors also show increased responsiveness to platinum containing drugs [32]. There are data suggesting that restoration of HR repair in a deficient background is a possible mechanism of resistance to these agents. In fact, reversion mutations or intragenic deletions in *BRCA1* and *BRCA2* mutated genes restored the protein reading frame resulting in a functional protein and the re-acquisition of HR proficiency [68,69,70,71] and a resistance to cisplatin and PARP inhibitors. Recently, *BRCA1* and *BRCA2* somatic mutations were detected in circulating free DNA of breast and ovarian tumors resistant to platinum-based therapy and to PARPi in patients with *BRCA1* and *BRCA2* germline mutations [62]. Again, these putative reversion mutations restored the protein reading frame possibly resulting in the reacquisition of the repair function of both BRCA 1 and 2 proteins. In addition, it was also reported that in a *BRCA1* mutated background, PARPi-induced resistance was associated with the ability to HSP90 to interact, stabilize, and re-render the mutant BRCA1 protein partially functional [72]. Indeed, the mutant BRCA1 protein was capable of promoting RAD51 loading onto DNA following DNA damage. Interestingly, it has been demonstrated that there was increased BRCA1 protein expression, in the absence of *BRCA1* reversion mutation, in two of four tumors carrying BRCT domain mutations in ovarian tumor samples resistant to carboplatin [72]. Secondary somatic mutations in *RAD51C* and *RAD51D* genes restoring the reading frame of the proteins have been reported in post-progression tumor samples obtained from patients participating in ARIEL2 Part1 clinical trial, in which patients with platinum-sensitive, relapsing tumor were treated with the PARP inhibitor rucaparib. These secondary mutations were associated with rucaparib resistance [73].

Recently data on the importance of the stabilization/resolution of the stalled replication fork have been shown to be associated with resistance to anticancer agents, including platinum containing drugs [34]. In particular, it has been demonstrated that BRCA1 and BRCA2, key players in HR repair, have also an HR-independent role in protection of the replicative fork by inhibiting the MRE11-dependent nucleolytic degradation of nascent DNA [74,75]. The loss of Pax transactivation-domain interacting protein (PTIP), PARP1, and Chromodomain Helicase DNA Binding Protein 4 (CHD4) in a *BRCA2* deficient background was associated to resistance to cisplatin and PARP inhibitors, not due to the re-acquisition of a functional HR, but to an increased protection of replication forks [74]. The same authors further demonstrated that platinum treated *BRCA1/2* mutated ovarian patients with high level of PTIP expression had a longer progression free survival than similar patients with lower PTIP expression level, suggesting PTIP is a biomarker for platinum resistance [74]. Replication fork protection due to replication protein A (RPA) has been recently put forward as a possible mechanism of cisplatin resistance [76]. The authors demonstrated that cisplatin sensitivity in a panel of HGSOC cell lines correlated with the inability to execute an efficient NER repair during the S phase of the cell cycle for RPA exhaustion. Indeed, ectopic expression of RPA overcame replication fork instability and could rescue cisplatin sensitivity.

Nucleotide excision repair is the pathway involved in the repair of platinum-induced intra-strand crosslinks as well as in the removal of inter-strand cross-links along with HR, FA, and translational repair. Nucleotide excision repair genes have been shown to be altered (non-synonymous, splice mutations, and homozygous deletions of NER genes) in about 8% of EOC and these NER alterations were associated with improved overall survival (OS) and progression-free survival (PFS), compared with patients without NER alterations. This suggests that NER pathway inactivation in EOC could confer platinum sensitivity, similar to *BRCA1/2*-mutated tumors. Interestingly, in vitro data showed that NER alteration did not confer sensitivity to PARP inhibitors agent, suggesting that sensitivity to platinum and PARP inhibitors does not always occur in parallel [77].

Among the NER proteins, *ERCC1* is the most studied as a possible predictive biomarker of platinum response in different tumor types, including ovarian carcinoma. The ERCC1 protein, complexed with XPF, is involved in the 5′cleavage of the DNA strands carrying the platinum adduct. However, the complex has also a role in homologous recombination taking part in the repair of the intra-strand cross-link [42]. At the protein level, conflicting results have been reported on the role of ERCC1, in predicting response to cisplatin in ovarian cancer. In some studies, a positive correlation was found while in others this correlation was absent or even negative [78,79,80,81,82,83]. The lack of a validated antibody working in immuno-histochemistry renders the interpretation of the different studies and their correlation with the response to a platinum-based therapy difficult [84]. Several reports tried to correlate the presence of gene polymorphisms that could somehow alter the expression and stability of coded protein in DNA repair genes, with again no clear results, often due to the limited number of patients present in each study. The *ERCC1* gene has been analyzed in depth for the presence of polymorphisms able to determine outcome of EOC patients. Again conflicting results have been reported, although an analysis of 10,000 patients failed to detect any correlation between polymorphisms and clinical outcome of EOC patients [85,86,87,88,89]. Other NER genes were studied, including *XPA*, *XPB*, *XPF*, and *XPD*, both at the mRNA level and gene polymorphisms, but none have been shown to be correlated with cisplatin resistance in ovarian carcinoma [82,86,90].

Interesting data have been reported on the role of DNA polymerase eta, a protein involved in translesion synthesis after 8-oxo-Guanine formation [91]. This protein encoded by the *PLH* gene, has been in fact shown to play a role in the repair of cisplatin-DNA adducts. Its expression seems to be higher in cancer stem cells, thus possibly accounting for the intrinsic resistance this subpopulation has to cisplatin treatment [92,93]. Potential inhibitors of polymerase eta have recently shown to potentiate cisplatin activity [94].

## 5. Functional Assays to Predict DNA Repair Proficiency

Recently, several attempts have been made to set up methods able to determine the functional activity of DNA repair pathways rather than the genetic alteration/s or differential expression of the specific genes involved in the pathways. The availability of these functional assays would allow a better evaluation of the cellular DNA repair capacity to be correlated with the sensitivity of a given drug and/or the acquisition of drug resistance. Some of these methods have been established and validated in in vitro systems, especially using isogenic cell lines, in which the only difference is a single key gene whose deletion and/or mutation determines the functional inactivation of the DNA repair pathway. Most of these functional assays rely on the in vitro or ex vivo induction of a specific DNA damage (i.e., by drug treatment) in order to induce DNA damage and elicit DNA repair, whose functionality will be assessed. However, the clinical transposition of such assays could be problematic as they require the use of ex vivo patient samples, which is not always available (i.e., lung biopsies) and comes with the risk of in vitro cell selection when using primary cultures. The ideal functional assay would be an assay to be performed at basal condition in formalin-fixed paraffin-embedded (FFPE) tumor samples. The other possibility is to perform therapeutic window opportunity neo-adjuvant trials in which a basal biopsy could be compared with biopsies obtained at the diagnosis and at de-bulking surgery after treatment.

As already pointed out, the NER pathway involves more than 30 proteins and one of the limiting steps in the process is the activity of the ERCC1/XPF endonuclease. It has been shown that the *ERCC1* gene codes for several isoforms but only one, quantitatively low expressed, is able to bind to XPF to form the active complex able to work in excision repair [84,95]. This could be the reason for the controversial results obtained by measuring total ERCC1 expression. New antibodies able to specifically recognize this isoform bound to ERCC4 or methods such as the proximity ligation assay (PLA) which determines the active ERCC1 small isoform/XPF complex, are being investigated as potential biomarkers [95,96]. The preclinical evidence suggest that the reported PLA method is able to detect the active ERCC1/XPF complex only in cells with a NER proficient pathway and this correlates with platinum sensitivity. We have preliminary data showing that in ovarian cancer patient xenografts made resistant in vivo to cisplatin, a higher level of ERCC1/XPF complex detected by PLA was found compared to platinum-sensitive xenografts [97]. These data need, however, to be validated in a larger cohort of patients with platinum-resistant tumors.

In the last decade, molecular analysis of the HR genes by next-generation sequencing and or whole genome sequencing has led to the development of tests (such as the BROCA test, Myriad and Foundation Medicine, and HRD detect) that, based on the presence of mutations in HR genes and genomic scars (i.e., loss of heterozygosity, large-scale transition, and telomeric imbalance), have provided the possibility to classify tumors as HR proficient or deficient (HRD) [98]. However, these tests have some limitations since they are not considering the epigenetic mechanisms of gene silencing leading to HR deficiency. In addition, the presence of a specific genomic scar only states that at a certain point the tumor had HRD, but this could not be the case at the time of tumor testing (i.e., reversion mutation in HR that re-confer a HR proficient phenotype). All these considerations have prompted the development of functional tests for HR repair, being one of most reliable the quantification of RAD51 foci formation after damage, generally i.e., ionizing radiation, whose lesions are repair by HR repair, using ex vivo experimental setting. The RAD51 foci formation test has been shown to predict the response to PARP inhibitors in ovarian cancer [99,100]. Given that HR is required for the repair of cisplatin-induced DNA damage, this could also be considered a predictor of platinum sensitivity. In addition, this functional assay has been shown to predict resistance to PARP inhibitors in a *BRCA1/2* germline background, demonstrating a re-activation of a functional HR as a mechanism of drug resistance. Very recently it was shown that not only the RAD51 foci formation after damage, but also the number of RAD51 foci (RAD51 score) in basal untreated samples correlated with PARP inhibitors response, with high RAD51 score predicting poor response and low RAD51 score good response [101]. In an attempt to develop predictive biomarkers of platinum response to primary chemotherapy, Tumiatti et al. [102] developed an HR score based on ex vivo scores of RAD51-possitive cells among the G2 cell fraction after IR damage and reportedly predicted chemotherapy response with high confidence in primary ovarian cancer and ascites. Interestingly enough, these authors corroborate data from others [103] that specimens from different locations of the same patients can display different DNA repair proficiencies, suggesting that resistance to chemotherapy, including platinum drugs, could be to the selection and overgrowth of DNA repair proficient clones. These data underscore the importance of intra-tumor heterogeneity as a cause of resistance to therapy.

Recently, a functional defect in replication fork protection was found to correlate with sensitivity to carboplatin in a panel of 33 organoid cultures derived from 27 patients with HGSOC [104]. The authors were able to set-up organoid cultures from fresh tumor samples that phenocopied the original tumors. Using this organoid platform, they applied the fiber assay as a read-out of the replication fork stability. They found that 91% of the organoids exhibiting stable forks were carboplatin resistant and 76% of the organoids with unstable fork were carboplatin sensitive. Similar results were obtained when the sensitivity to Chk1 and ATR inhibitors were looked for. These data suggest that replication fork stability could be a predictor of response/resistance to specific DNA interfering agents, even if its predictive role needs to be validated in a larger cohort of tumor samples/organoids.

## 6. Models to Study Drug Resistance in Ovarian Cancer

To better understand the molecular mechanisms and the players determining drug resistance in ovarian cancer, it is mandatory to have preclinical models recapitulating the clinical setting. The results coming from these models could be then tested in the clinical specimen as potential biomarkers.

Several two-dimensional human or murine ovarian cancer cell lines are available. Some of them have been made resistant in vitro to cisplatin by treating cells with increased concentrations of the drugs. These cells have been useful in highlighting some of the mechanisms of resistance, including those related to DNA repair [55,105,106,107,108,109,110]. These systems, although not always being able to give results translatable to the clinic, are useful in setting up methods, as already discussed, such as functional assays to measure DNA repair, which could be improved and adapted to the clinic.

The development of models more closely related to the tumors of the patients, such as patient-derived xenografts (PDXs), have been developed [111,112,113,114,115]. These models, although not always suitable to study fine mechanisms of drug sensitivity or resistance, are unique tools to study in a more predictable way the potential benefits of biomarkers. In our laboratory we have generated a EOC xenobank consisting of more than 60 PDXs whose characteristics well recapitulate the tumor they originate from [111]. This xenobank includes few intrinsically platinum-resistant tumors, which offer the possibility to study in detail the mechanisms of intrinsic resistance. The majority of the PDXs present in the xenobank, however, initially respond to platinum-based chemotherapy, again similar to what is observed in the clinic. Treating relapsing tumors in mice with subsequent cycles of platinum-based therapies, led to the development of resistant tumors (directly obtained in vivo) which can now be used to understand the molecular mechanisms as the basis of resistance and to test new therapies and or combinations [116]. Initial studies aimed at defining the importance in DNA repair in mediating responsiveness to therapy, identified several interesting correlations among the expression of genes belonging to different pathways [117]. In particular it was found that the mRNA levels of CDK12 associate with high recurrence rates in these PDX, again supporting a master role of this CDK in controlling DNA repair [117]. These sensitive/resistant sublines are currently used to set up in vivo functional DNA repair assays. Sensitive and resistant PDXs were also used to test new second line therapies which could maintain effectiveness in platinum resistant tumors [118].

While PDXs represent important pre-clinical models that helped provide better understanding of the biology and the development of new therapeutic strategies, they are time-consuming and costly. Recently the use of organoids derived from different tumor type have been suggested as an intermediate tool between 2D cultures in vitro and PDXs [119,120,121]. They maintain important characteristics of the tumors they originate from together with infiltrating cells [122,123,124,125]. These organoids, therefore, present an increased complexity relative to 2D cultures yet allow high throughput studies at present not possible in PDXs [120,122,126]. As already discussed, these organoids have been generated also for ovarian cancer, and in the future, more of these models are likely to be used for functional tests [104].

## 7. Conclusions

In spite of the increasing knowledge of the biology of tumors, including EOC, the use of “old” chemotherapeutic drugs still remains the first option for patients with cancer. This is particularly true for EOC, where first line treatment invariably includes platinum-based chemotherapy. The majority of EOCs respond to platinum-containing drugs, but unfortunately, often relapse with a tumor no more (or less) responsive to these drugs. The development of acquired resistance remains one of the major obstacles to a true increase in OS for patients with EOC. Platinum-containing drugs act mainly to induce DNA damage which activates a DNA damage response. Several mechanisms of resistance associated to changes in DNA repair have been discussed in this review. We still lack a powerful biomarker able to predict the resistance in advance as well as ways to counteract this phenomenon. New inputs have been produced by the discovery that DNA damage defective tumors (such as *BRCA1* and *BRCA2* carriers for HR), which can be particularly susceptible to inhibitors of other DNA repair systems such as PARP inhibitors. The availability of new functional assays for DNA repair and new tools to study both in vitro and in vivo the mechanisms at the basis of drug resistance will certainly and hopefully impact future patients’ outcome.

## Figures and Tables

**Table 1:** Major pathways involved in the repair of platinum DNA damage.

Platinum DNA Damage	Repair Pathway	Specific Genes Implicated in Platinum Resistance
Mono-adduct	Base excision repair (BER)	*OGG1*, *PARP1*
Intra-strand cross-link	Nucleotide excision repair (NER) Mismatch repair (MMR) Tolerance pathway (translesion synthesis)	*ERCC1*, *XPF*, *XPD*, *XPG* *MLH1, MSH2, MSH6, PMS2* Polymerase (pol) η, ζ Rev1
Inter-strand cross-link	NER Famconi Anemia (FA) Homologous recombination (HR)	*ERCC1*, *XPF*, *XPG*, *XPD* FA core complex genes, *FANCD2* *BRCA1*, *BRCA2*, *RAD51*, *RAD52*, *XRCC2*, *XRCC3*, *RPA*
Double-strand break	HR Non-homologous end joining (NHEJ)	*BRCA1*, *BRCA2*, *RAD51*, *RAD52*, *XRCC2*, *XRCC3*, *RPA* *XRCC4*, *XRCC5*, *KU70*, *DNA-PKcs*
